# Dietary and Smoking Acrylamide and Prostate Cancer Risk: CAPLIFE Study

**DOI:** 10.3390/nu16060836

**Published:** 2024-03-14

**Authors:** Macarena Lozano-Lorca, Carlos Muñoz-Bravo, Rocío Barrios-Rodríguez, María Ángeles Castillo-Hermoso, Malak Kouiti, Carla González-Palacios Torres, José-Juan Jiménez-Moleón, Rocío Olmedo-Requena

**Affiliations:** 1 Departamento de Medicina Preventiva y Salud Pública, Universidad de Granada, 18016 Granada, Spain; macarenalozano@ugr.es (M.L.-L.); rbarrios@ugr.es (R.B.-R.); mariacasherm@correo.ugr.es (M.Á.C.-H.); m.kouiti@uhp.ac.ma (M.K.); carlagpt@ugr.es (C.G.-P.T.); jjmoleon@ugr.es (J.-J.J.-M.); rocioolmedo@ugr.es (R.O.-R.); 2 Instituto de Investigación Biosanitaria ibs.GRANADA, 18014 Granada, Spain; 3 Centro de Investigación Biomédica en Red de Epidemiología y Salud Pública, Instituto de Salud Carlos III, 28029 Madrid, Spain; 4 Department of Public Health and Psychiatry, School of Medicine, University of Málaga, 29071 Málaga, Spain; 5 Biomedical Research Institute of Malaga (IBIMA), 29010 Málaga, Spain; 6 Laboratory of Health Sciences and Technologies, Higher Institute of Health Sciences, Hassan First University of Settat, Settat 26000, Morocco

**Keywords:** acrylamide, case–control studies, diet, prostate cancer, smoking

## Abstract

Acrylamide is a probable carcinogen. Its main sources are the diet and tobacco. The association between acrylamide intake from the diet and tobacco and prostate cancer (PCa) has not been previously evaluated. We aimed to evaluate the relationship between dietary acrylamide intake and exposure to acrylamide through cigarettes and PCa risk. A population-based case–control (CAPLIFE) study was conducted, including 428 incident PCa cases and 393 controls. Smoking and dietary information, with a validated food frequency questionnaire, was collected. We calculated the amount of acrylamide from both sources, and tertiles (Ts) were created. Multivariable logistic regression and restricted cubic spline models were applied to assess the association between exposure to acrylamide and PCa risk. The median was similar for acrylamide in both dietary and smoking acrylamide among PCa cases and controls. No association was observed between dietary acrylamide intake and overall PCa risk (adjusted OR_T3vsT1_ = 0.90 (95% CI 0.59, 1.37)). A risk trend was observed for acrylamide exposure from cigarette smoking (*p*-trend = 0.032), with the highest odds in those subjects with the high exposure to acrylamide through cigarettes (adjusted OR_T3vsT1_ = 1.67 (95% CI 0.92, 3.04)). The restricted cubic splines suggested a linear relationship. In conclusion, acrylamide from smoking could be positively associated with PCa risk, but no association was observed for dietary acrylamide.

## 1. Introduction

Acrylamide is a colorless, odorless, crystalline compound [1]. Its low molecular weight allows it to be easily distributed through the tissues, and it is eliminated mainly through the urine [2]. Currently, the two known main sources of acrylamide exposure are exogenous; these are tobacco smoke [3,4] and heat-treated foods, especially in carbohydrate-rich foods such as potatoes, cereal-based products, and coffee [5]. In addition, it has been suggested by in vivo studies that acrylamide is possibly also formed endogenously due to oxidative stress [6].

There is evidence that acrylamide has adverse health effects, including neurotoxicity [7,8] and impaired fertility [9]. Moreover, experimental studies in rodents point to a possible carcinogenicity of this substance, as indicated by the Scientific Opinion on acrylamide in food of the European Food Safety Authority (EFSA) in 2015 [10]. In 1994, the International Agency for Research on Cancer (IARC) declared acrylamide as a probable human carcinogen (Group 2A) [11]. Despite this, in recent decades, in Europe, there has been an increased trend of exposure to this substance, measured by acrylamide biomarkers [12]. In this sense, with the aim of mitigating the concentration of acrylamide in the food chain, the European Union Commission Regulation 2017/2158 implemented a series of measures fundamentally based on the temperature and time of cooking/frying/roasting.

It was in 2002 that the presence of acrylamide in the diet was identified for the first time, leading to worldwide concern [5]. Since then, various studies have quantified the amount of acrylamide ingested through the diet and its relationship with, mainly, colorectal [13,14], breast [15,16], ovarian, and endometrium cancer [17], among others. However, as recognized by Hogervorst et al. in a recent editorial about dietary acrylamide and human cancer, “the body of evidence is still cloudy” [18]. For prostate cancer (PCa) specifically, previous studies have not found an association between acrylamide intake and the risk of this tumor [19,20,21,22,23,24,25,26,27]. Nevertheless, these studies considered only diet as the source of exposure.

PCa is the leading cancer type among men in Europe, and an increase in its incidence has been observed in recent decades [28]. Furthermore, PCa is known to be a heterogeneous disease in terms of aggressiveness, extension, and prognosis [29], and the role of acrylamide could be affected by the stage and/or grade of the tumor; however, few studies have considered these aspects in the association between dietary acrylamide intake and PCa [19,23,24,25]. 

Given the tendency towards increased exposure to acrylamide in recent decades, the lack of studies that address exposure to acrylamide through its two main sources (diet and smoking) in the same population regarding the risk of PCa, the increase in the incidence of this tumor, and the scarce consideration of stage and grade of PCa, this study aimed to evaluate the relationship of dietary acrylamide intake and exposure to acrylamide from cigarette smoking with PCa risk, considering the tumor grade and stage.

## 2. Materials and Methods

### 2.1. Study Design

The CAPLIFE study is a population-based case–control study whose general aim is to evaluate the association between lifestyles and PCa risk. The participant recruitment was carried out in the two main university hospitals in Granada (Spain), Virgen de las Nieves and Clínico San Cecilio Hospital, between May 2017 and September 2020. Previous studies have extensively described the methodology of the CAPLIFE study [30,31,32]. 

### 2.2. Participants

The criteria for selection for PCa cases and controls were as follows: (1) age between 40 and 80 years; (2) residence in the coverage area of the reference hospitals for at least 6 months; and (3) new diagnosis of PCa with histopathological confirmation before receiving treatment (only for PCa cases) as defined by the International Classification of Diseases and Related Health Problems 10th Revision (ICD-10): C61 [33].

Cases were recruited and invited to participate in the urology services through the pathological anatomy lists. Controls were chosen at random from the patient lists of general practitioners of 16 primary healthcare centers belonging to the Granada-Metropolitan Health District. For its recruitment, the age at diagnosis of PCa (±5 years) was considered based on the information obtained from the Granada Cancer Registry. Controls were selected by density sampling over time. 

### 2.3. Data Collection

Information was collected using a structured computerized questionnaire conducted face to face by trained interviewers and from participants’ medical history.

#### 2.3.1. Dietary Acrylamide

A self-administered food frequency questionnaire (FFQ) previously validated for the Spanish population was used for the collection of dietary information [34,35]. The FFQ referred to the 12 months prior to the interview and collected information on the usual consumptions of 134 foods, including regional products (typically produced and consumed in Spain), and beverage items with portion sizes specified for each item. The frequency response choices were from “never” to “more than 6 times a day”. 

To determine the dietary acrylamide intake of each participant, first, 20 of the 134 items in the FFQ that are acrylamide-containing were selected. These items were classified into 6 food groups in a similar way to the Bellicha et al. study [16]: (i) potato fries and chips; (ii) bread; (iii) breakfast cereals; (iv) mixed dishes (pasta, pizza, and croquettes); (v) cookies, pastries, and chocolates; and (vi) coffee. For the determination of the concentration of acrylamide in these 20 acrylamide-containing foods, the quantity of acrylamide for each food was derived from data published using Spanish populations, given the absence of an acrylamide database in this country [36,37,38,39,40,41,42,43]. If no previous Spanish study had evaluated the acrylamide concentration of a particular food, the Scientific Opinion on acrylamide in food of the EFSA was consulted [10]. The concentration of acrylamide in each food (µg/kg) and the source of information can be consulted in Supplementary Table S1. Next, we calculated daily acrylamide intake (µg/day) by multiplying the concentration of acrylamide in each food by the frequency of consumption and portion size. Finally, the amount of daily acrylamide from all foods was added. In addition, participants were categorized into tertiles using the control group cut-off points. Participants with implausible total energy intake were excluded (less than 800 kcal/day or more than 4000 kcal/day) [44]. 

#### 2.3.2. Smoking Acrylamide

Data on smoking status (never, former, or current smoker), years of smoking, and average number of cigarettes per day were considered for former and current smokers. The acrylamide from cigarette smoking (µg/day) was calculated by multiplying the average number of cigarettes per day by the average amount of acrylamide contained in each cigarette (566 ng/cigarette) according to Esposito et al. [45]. They randomly selected 65 conventional cigarettes in Italy, including different brands and types of cigarettes. Finally, participants were categorized into tertiles using the control group cut-off points of exposure to acrylamide from cigarette smoking.

#### 2.3.3. Measurement of Clinical Characteristics

For PCa cases, information on the aggressiveness and stage of the tumor was collected from medical records. For aggressiveness, the International Society of Urological Pathology (ISUP) grade (from 1 to 5; a higher degree is indicative of greater aggressiveness) was used. Specifically, the ISUP grade was categorized as follows [46,47]: ISUP 1–2 (low aggressiveness) and ISUP 3–5 (high aggressiveness). Regarding staging, the clinical Tumor, Node, Metastasis (TNM) classification was used to classify tumors in two categories: i) localized and ii) locally advanced (cT3/cT4 or N1) or metastatic (M1) [48]. Both the aggressiveness classification and staging systems are strongly recommended by the European Association of Urology (EAU). 

#### 2.3.4. Sociodemographic Data and Personal History

Information on sociodemographic data (age, education level, and marital status), body mass index (BMI), first-degree family history of PCa, other lifestyle factors (physical activity, sedentary behavior), and self-reported diabetes mellitus (type 1 or 2) was also collected. Physical activity and sedentary behavior information were collected through the International Physical Activity Questionnaire Short-Form (IPAQ) [49].

### 2.4. Statistical Analysis

Characteristics were described using the median and interquartile range (IQR) for the continuous variables and the distribution of absolute and relative frequencies for the categorical variables. The Mann–Whitney U or Kruskal–Wallis tests were used to assess the level of significance of the differences observed in the continuous variables. For categorical variables, the chi-squared test was conducted.

Multivariable logistic regression models were used to estimate adjusted odds ratios (aORs) and 95% confidence intervals (95% CIs) for both the association between the tertiles (Ts) of dietary acrylamide intake and the association between tertiles of acrylamide from cigarette smoking and PCa. For both variables, the first tertile (lower exposure) was considered the reference category. In addition, the association between the combination of tertiles from both sources (diet and cigarettes), as a single variable, and the risk of PCa was calculated. We used information from previous studies and a directed acyclic graph (DAG) to identify potential confounders; thus, epidemiological, and statistical criteria were used to construct the models. Potential confounding factors included design-related variables (age and educational level) and variables that the scientific literature had linked to PCa (first-degree family history of PCa, smoking status, BMI, physical activity, and diabetes mellitus) and, at the same time, they were statistically related to acrylamide (*p* < 0.20) (Supplementary Tables S2 and S3). Therefore, the models included the following adjustment variables: age, educational level, first-degree family history of PCa, smoking status, BMI, physical activity, and diabetes mellitus. The model for dietary acrylamide intake was also adjusted for energy intake. Additional models were run, mutually adjusting for the other exposure variables, dietary acrylamide intake, or acrylamide from cigarette smoking, as appropriate. Logistic regression models with restricted cubic splines were used to address the potential nonlinearity of the association between both continuous variables (dietary and smoking acrylamide) and PCa risk. In addition, analyses were stratified according to ISUP grade and staging of the tumor. 

All statistical tests were two-sided, and statistical significance was set at *p* < 0.05. Statistical analyses were performed using the statistical program Stata v.15 (Stata Corp., 2017, College Station, TX, USA).

## 3. Results

**Figure 1:**
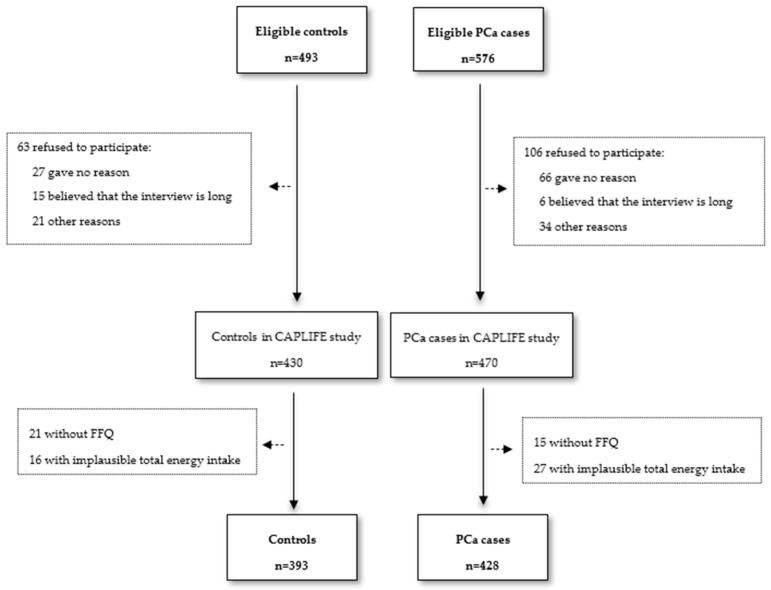
Flowchart of CAPLIFE study.

A sample of 428 cases and 393 controls was included in the analysis (Figure 1). The characteristics of PCa cases and controls are shown in Table 1. The median of dietary acrylamide intake was 18.9 µg/day (IQR 13.3–31.1) for cases and 19.2 µg/day (IQR 13.5–29.5) for controls. Regarding exposure to acrylamide from cigarette smoking, this was 8.5 µg/day (IQR 0.0–11.6) for cases and 8.5 µg/day (IQR 0.0–14.2) for controls—for current smokers, the median was 11.3 µg/day (IQR 7.1–14.2) in the PCa case group and 10.7 µg/day in the control group (IQR 8.0–11.3). PCa cases were significantly older, presented greater energy intakes, and more frequently had a first-degree family history of PCa than controls (*p*-value < 0.05). Most cases had an ISUP 1–2 tumor (75.2%), and this tumor was localized (86.2%). The characteristics of controls and PCa cases across tertiles of acrylamide from the diet and cigarette smoking are shown in Supplementary Tables S2 and S3.

**Figure 2:**
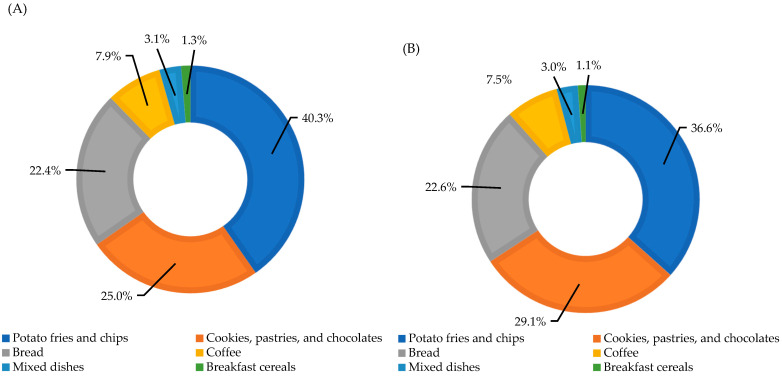
The relative contribution of the six food groups to acrylamide intake (%) for controls (**A**) and PCa cases (**B**).

Figure 2 shows the contribution of the six food groups to acrylamide intake (%) for controls (Figure 2A) and PCa cases (Figure 2B). The main contributors to acrylamide intake were potato fries and chips (36.6% of the amount of acrylamide ingested for cases and 40.3% for controls), cookies, pastries, and chocolates (29.1% and 25.0% for cases and controls, respectively), bread (22.6% for cases and 22.4% for controls), and coffee (7.5% and 7.9% for cases and controls, respectively). Overall, these four food groups contributed more than 90% of total acrylamide intake both in cases and controls.

**Figure 3:**
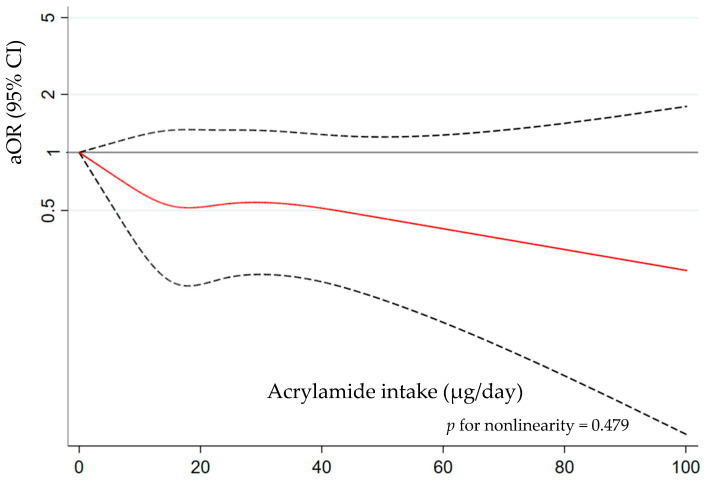
Approximated nonlinear trend between acrylamide intake and overall prostate cancer (PCa) risk using restricted cubic spline. Data are odds ratios and 95% confidence intervals [aOR (95% CI)] adjusted for age, educational level, first-degree family history of PCa, smoking status, body mass index, physical activity, diabetes mellitus, and energy. Note: dashed lines indicate the 95% confidence intervals and the red solid line indicates the point estimate of aORs.

The association between tertiles of dietary acrylamide intake and PCa risk is shown in Table 2. No association was found between the acrylamide intake and the overall risk of PCa (aOR_T3vsT1_ = 0.90 (95% CI 0.59, 1.37)), neither when stratifying by ISUP grade and tumor stage. The nonlinear association analysis was not significant (*p* for nonlinearity = 0.479) (Figure 3), neither for the different ISUP grades and tumor stages (Supplementary Figure S1), which is indicative of a linear association. A sensitivity analysis was carried out by adjusting the acrylamide intake in the total energy intake using the residual method and the results did not change.

**Figure 4:**
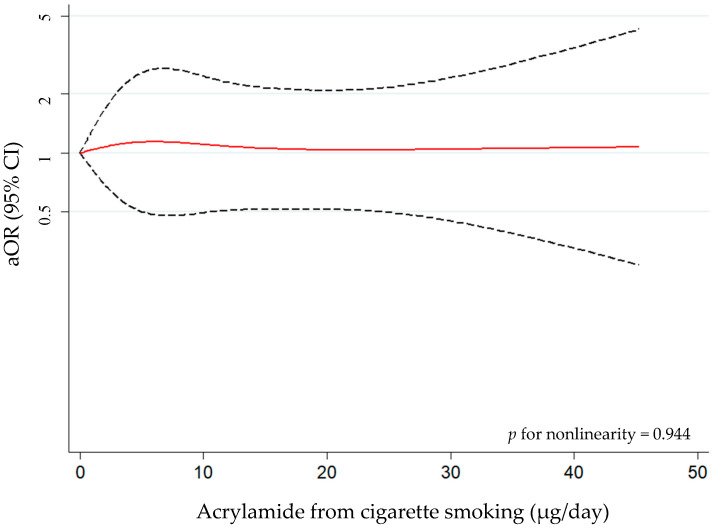
Approximated nonlinear trend between acrylamide from cigarette smoking and overall prostate cancer (PCa) risk using restricted cubic spline. Data are odds ratios and 95% confidence intervals [aOR (95% CI)] adjusted for age, educational level, first-degree family history of PCa, smoking status, body mass index, physical activity, and diabetes mellitus. Note: dashed lines indicate the 95% confidence intervals and the red solid line indicates the point estimate of aORs.

Table 3 shows the association between the tertiles of acrylamide from cigarette smoking and PCa. A risk trend was observed for overall PCa cases (*p*-trend = 0.032). Those subjects with greater exposure to acrylamide through cigarettes (T3) presented the highest odds of PCa [aOR_T3vsT1_ = 1.67 (95% CI 0.92, 3.04)]. This risk trend was maintained for ISUP grade 1–2 [aOR_T3vsT1_ = 1.61 (95% CI 0.85, 3.03)] and localized tumors [aOR_T3vsT1_ = 1.75 (95% CI 0.94, 3.26)]. The nonlinear association analysis was not significant (*p* for nonlinearity = 0.944) (Figure 4), which is indicative of a linear association. By combining the tertiles from both sources, we cannot establish that there is a linear trend between them and the risk of PCa (*p*-trend = 0.172) (Supplementary Table S4).

## 4. Discussion

To our knowledge, this is the first study to incorporate the assessment of tobacco as a major source of acrylamide exposure, along with diet, and its association with PCa risk. The main contributors of acrylamide in diet were the following four groups: (1) potato fries and chips; (2) cookies, pastries, and chocolates; (3) bread; and (4) coffee. No association was found between dietary acrylamide intake and overall PCa. However, a risk trend was observed for exposure to acrylamide from cigarette smoking. Restricted cubic spline analyses suggested a linear relationship between acrylamide from cigarettes smoking and PCa.

Acrylamide was classified as a probable human carcinogen (Group 2A) by IARC in 1994 [11]. Since then, no changes have been produced for this classification according to new knowledge. Three systematic reviews and meta-analyses have recently been published with the objective of evaluating the association between dietary acrylamide exposure and several site-specific cancers with inconclusive results [26,50,51]. There are several mechanisms that could explain the possible carcinogenicity of this substance: (i) glycidamide, an epoxide metabolite of acrylamide, which forms DNA adducts [52,53]; (ii) acrylamide has been related to alteration of sex hormones [54]; and (iii) acrylamide-induced oxidative stress [55]. Individual exposure to acrylamide can be investigated from acrylamide concentrations in food, tobacco exposure, or through human biomonitoring and analysis of biomarkers using biological sources such as blood [56] or urine [57], an objective method that requires laborious sampling work. Although the limitations of the estimation of food components from FFQs are well-known (complex elaboration, requires memory, and can overestimate intake) [58], it allows the evaluation of past exposures, while human biomonitoring usually allows quantifying short-term exposure levels. 

The exposure to dietary acrylamide is very variable in the literature. Our values were similar to those reported in previous epidemiological studies that analyzed the association between acrylamide and the risk of PCa [19,22,24]. However, other studies with the same objective report slightly higher or lower acrylamide levels. Thus, the cohort study carried out in Japan reported lower acrylamide intake (mean of the cohort: 6.4 (SD 2.0) µg/day) [20] and three previous studies reported higher levels [median for Finnish cohort study: 36.7 µg/day; mean for cases and controls of Sweden study: 43.8 µg/day (SD 13.7) and 44.5 µg/day (SD 14.5), respectively; and median for Swedish cohort study: 35.4 µg/day] [21,23,25]. These differences in the levels of acrylamide may be due to: (1) a large difference in diet between places. In this way, the foods considered in each study varies. Specifically, these studies reporting higher intakes of acrylamide include crisp rye bread, a food that contains large amounts of acrylamide but is not part of the usual diet of the Spanish population [21,23,25]; (2) the concentration of acrylamide in each food differs depending on the source consulted. In our study, those indicated in Spanish publications were used, and failing that, the one reported by the Scientific Opinion on acrylamide in food of the EFSA [10]. Nevertheless, the food groups with a higher contribution to the acrylamide intake in our study were similar to those previously published, although most of them place coffee as the main or secondary source of acrylamide [19,21,23,24,25]. 

No association was found between the dietary acrylamide intake and overall PCa risk. This result is consistent with those found in previous studies [19,20,21,22,23,24,25]. In line with our results, the three cohort and one case–control studies that considered the staging of the tumor in the association between dietary acrylamide and PCa also found no association regardless of staging [19,23,24,25]. This fact may be due to: (i) there is no association between exposure to dietary acrylamide and the risk of PCa; or (ii) the existence of a non-differential bias towards the null that equally affects PCa cases and controls. This possible bias could be due to a mismeasurement of the diet, the estimation of the mean dietary acrylamide ingested from the estimated mean for each food, although the amount of acrylamide can vary widely depending on the degree of cooking or brand. 

Regarding the exposure to acrylamide from cigarette smoking, we observed a risk trend for PCa. This risk trend was maintained for ISUP grade 1–2 and localized tumors. These results are interesting when considering that smokers have been observed to present 2.5 times higher levels of acrylamide metabolite in urine than non-smokers [59]. We failed in comparison with other studies because, to date, efforts have been focused on analyzing the association between dietary acrylamide and different tumors [16,20,60], but no previous study has analyzed the relationship between acrylamide in tobacco and the risk of developing cancer.

To our knowledge, this is the first study to incorporate the assessment of tobacco as a major source of acrylamide exposure, along with diet.

Our study has several strengths: (i) to our knowledge, no previous study has quantified exposure to acrylamide in tobacco and its association with PCa, and therefore this is the first study to include both sources (diet and smoking) and evaluate the association with PCa risk, considering ISUP grade and stage of the tumor; (ii) the use of a semi-quantitative FFQ for the evaluation of the diet. It is a validated questionnaire that includes regional products and is recognized as a standard method for assessing habitual dietary intake [34,35]. Moreover, although the FFQ refers to the previous year, dietary habits have been shown to remain fairly stable over time [61]; (iii) the number of foods considered in the dietary exposure to acrylamide was higher than most previous studies [19,22,23,24,25], including typical foods in Spain such as churros or polvorones. For these reasons, we consider that the estimate of dietary acrylamide should be very close to what is ingested by our population; and (iv) for estimation of acrylamide from cigarette smoking, we considered the different types and brands of cigarettes [45].

Some limitations may affect the results found. Regarding exposure to dietary acrylamide: (i) we could not take into account the modalities of preparation and cooking of foods (particularly temperature and duration), and the different product brands, aspects that condition the amount of acrylamide in food [10]; (ii) the possibility of recall bias in the collection of dietary information, as previously has been mentioned. However, a validated questionnaire, FFQ, was used to try to minimize it. We consider that if there were recall bias, it would have affected cases and controls in the same way, thus being a non-differential bias. Regarding exposure to acrylamide from cigarette smoking and PCa and the association with PCa, it is worth noting: (i) the sample size, we might have lacked statistics power to detect significant associations; (ii) the estimation of acrylamide from smoking was made from cigarettes and no other types of tobacco such as cigars, pipes or electronic cigarettes, as we have not found previous studies that report the amount of acrylamide in these others. Moreover, considering that the induction periods of cancer risk factors are very long, we may not be able to observe the results of new tobacco trends, such as electronic cigarettes, until after many years. However, taking this into account, it would be interesting for future studies to consider these exposure sources. Only 14 controls (3.6%) and 26 PCa cases (6.1%) reported having smoked pipes or cigars; (iii) we were unable to estimate acrylamide exposure from passive smoking. For both sources of exposure, we cannot rule out the possibility of residual confounding, although we adjusted for a range of potential confounders that were associated with both acrylamide exposure and PCa risk.

## 5. Conclusions

In conclusion, we found no evidence that dietary acrylamide intake is associated with overall PCa risk, nor when stratifying by ISUP grade and tumor stage. However, a risk trend was observed for exposure to acrylamide from cigarette smoking. Therefore, more studies are necessary to confirm the possible relationship between acrylamide in tobacco and PCa. The etiology of PCa is not well known. The study of the causes of PCa is very complex, as it is a very heterogeneous disease. It must be considered that the use of observational studies to establish causal relationships is limited due to multiple complicated and intractable issues. On the other hand, acrylamide is a substance to which we can be exposed from various sources. Therefore, more studies, preferably prospective, are necessary to confirm whether there is an association between acrylamide consumption, especially via tobacco, and the risk of PCa, including a good definition of exposure, working with incident cases of PCa and considering the different and new forms of tobacco consumption.

## Figures and Tables

**Table 1:** Characteristics of controls and prostate cancer (PCa) cases in the CAPLIFE study.

	Controls *n* = 393	PCa Cases *n* = 428	*p*-Value
Dietary acrylamide intake (µg/day), median (IQR)	19.2 (13.5–29.5)	18.9 (13.3–31.1)	0.717
Acrylamide from cigarette smoking ^a^ (µg/day), median (IQR)	8.5 (0.0–14.2)	8.5 (0.0–11.6)	0.638
Age (years), median (IQR)	66.5 (61.3–72.3)	68.5 (62.8–73.9)	0.005
Education level, n (%)			0.377
Primary	109 (27.8)	124 (28.9)	
Secondary	199 (50.6)	228 (53.3)	
University	85 (21.6)	76 (17.8)	
Marital status, n (%)			0.760
Married	330 (83.9)	356 (83.2)	
Not married	63 (16.1)	72 (16.8)	
Smoking status, n (%)			0.794
Never smoker	103 (26.2)	112 (26.2)	
Former smoker	219 (55.7)	231 (53.9)	
Current smoker	71 (18.1)	85 (19.9)	
Years of smoking, median (IQR)	35.6 (22.0–45.0)	37.0 (27.0–45.0)	0.478
Number of cigarettes per day, median (IQR) ^a^	20.0 (10.0–30.0)	20.0 (10.0–30.0)	0.564
Body mass index, n (%)			0.758
Normal weight	72 (18.4)	87 (20.4)	
Overweight	207 (52.6)	219 (51.3)	
Obesity	114 (29.0)	121 (28.3)	
Missing	–	1	
Physical activity, n (%)			0.158
Low	133 (33.8)	166 (38.7)	
Moderate	201 (51.2)	214 (50.0)	
High	59 (15.0)	48 (11.3)	
Sedentary behavior (h/day), median (IQR)	7.0 (5.0–9.0)	7.0 (5.0–10.0)	0.268
Energy intake (Kcal/day), median (IQR)	2290.3 (1943.1–2809.4)	2428.9 (2057.5–2918.9)	0.021
Alcohol consumption (g/day), median (IQR)	7.3 (1.4–15.9)	8.0 (1.5–18.5)	0.349
Diabetes mellitus, n (%)			0.364
No	299 (76.3)	337 (78.9)	
Yes	93 (23.7)	90 (21.1)	
Missing	1	1	
First-degree family history of PCa, n (%)			<0.001
No	350 (89.1)	333 (77.9)	
Yes	43 (10.9)	94 (22.1)	
Missing	–	1	
ISUP grade ^b^, n (%)			–
1–2	–	321 (75.2)	
3–5	–	106 (24.8)	
Staging of PCa, n (%)			
Localized	–	369 (86.2)	
Locally advanced–metastatic	–	59 (13.8)	

IQR, interquartile range (25th percentile–75th percentile); ISUP: International Society of Urological Pathology. ^a^ This information was available for a total of 388 controls and 425 PCa cases. ^b^ One subject could not be categorized using the ISUP classification as they had a neuroendocrine carcinoma.

**Table 2:** Association between tertiles of dietary acrylamide intake and overall prostate cancer (PCa) incidence, according to International Society of Urological Pathology (ISUP) grade and stage of PCa.

		Tertiles of Dietary Acrylamide Intake ^a^			
		T1	T2	T3	*p*-Trend
Overall	Controls/PCa cases	131/138	131/137	131/153	
	aOR (95% CI) ^b^	Reference	0.89 (0.62, 1.29)	0.89 (0.59, 1.36)	0.599
	aOR (95% CI) ^c^	Reference	0.91 (0.63, 1.32)	0.90 (0.59, 1.37)	0.627
ISUP grade					
1–2	Controls/PCa cases	131/103	131/103	131/115	
	aOR (95% CI) ^b^	Reference	0.92 (0.62, 1.36)	0.98 (0.62, 1.55)	0.939
	aOR (95% CI) ^c^	Reference	0.93 (0.63, 1.39)	0.99 (0.63, 1.57)	0.972
3–5	Controls/PCa cases	131/34	131/34	131/38	
	aOR (95% CI) ^b^	Reference	0.78 (0.43, 1.43)	0.63 (0.31, 1.27)	0.198
	aOR (95% CI) ^c^	Reference	0.80 (0.44, 1.47)	0.63 (0.31, 1.27)	0.195
Stage of tumor					
Localized	Controls/PCa cases	131/121	131/110	131/138	
	aOR (95% CI) ^b^	Reference	0.81 (0.55, 1.19)	0.94 (0.61, 1.44)	0.762
	aOR (95% CI) ^c^	Reference	0.82 (0.56, 1.21)	0.94 (0.61, 1.45)	0.771
Locally advanced–metastatic	Controls/PCa cases	131/17	131/27	131/15	
	aOR (95% CI) ^b^	Reference	1.49 (0.72, 3.09)	0.58 (0.22, 1.53)	0.329
	aOR (95% CI) ^c^	Reference	1.59 (0.76, 3.32)	0.62 (0.23, 1.66)	0.400

ISUP: International Society of Urological Pathology. ^a^ Acrylamide intake (µg/day) tertile cut-off points: T1: ≤15.0; T2: >15.0 to ≤24.3; and T3: >24.3. ^b^ aOR: Odds ratio adjusted for age, educational level, first-degree family history of PCa, smoking status, body mass index, physical activity, diabetes mellitus, and energy. ^c^ aOR: Odds ratio adjusted for age, educational level, first-degree family history of PCa, smoking status, body mass index, physical activity, diabetes mellitus, energy, and acrylamide from cigarette smoking.

**Table 3:** Association between acrylamide from cigarette smoking and overall prostate cancer (PCa) incidence, according to International Society of Urological Pathology (ISUP) grade and stage of PCa.

		Tertiles of Acrylamide from Cigarette Smoking ^a^			
		T1	T2	T3	*p*-Trend
Overall	Controls/PCa cases	133/140	150/150	105/135	
	aOR (95% CI) ^b^	Reference	1.19 (0.66, 2.13)	1.67 (0.92, 3.04)	0.031
	aOR (95% CI) ^c^	Reference	1.19 (0.66, 2.13)	1.67 (0.92, 3.04)	0.032
ISUP grade					
1–2	Controls/PCa cases	133/99	150/114	105/105	
	aOR (95% CI) ^b^	Reference	1.09 (0.58, 2.02)	1.61 (0.85, 3.04)	0.039
	aOR (95% CI) ^c^	Reference	1.08 (0.58, 2.02)	1.61 (0.85, 3.03)	0.041
3–5	Controls/PCa cases	133/41	150/35	105/30	
	aOR (95% CI) ^b^	Reference	1.47 (0.53, 4.05)	1.92 (0.69, 5.37)	0.177
	aOR (95% CI) ^c^	Reference	1.48 (0.54, 4.07)	1.93 (0.69, 5.39)	0.177
Stage of PCa					
Localized	Controls/PCa cases	133/114	150/133	105/120	
	aOR (95% CI) ^b^	Reference	1.22 (0.66, 2.23)	1.75 (0.94, 3.26)	0.024
	aOR (95% CI) ^c^	Reference	1.22 (0.66, 2.23)	1.75 (0.94, 3.26)	0.025
Locally advanced–metastatic	Controls/PCa cases	133/26	150/17	105/15	
	aOR (95% CI) ^b^	Reference	0.98 (0.29, 3.32)	1.30 (0.38, 4.43)	0.528
	aOR (95% CI) ^c^	Reference	0.98 (0.29, 3.32)	1.30 (0.38, 4.45)	0.523

ISUP: International Society of Urological Pathology. ^a^ Acrylamide from cigarette smoking (µg/day) was available for a total of 388 controls and 425 PCa cases, and the tertile cut-off points were: T1: ≤2.3; T2: >2.3 to ≤11.3; and T3: >11.3. ^b^ aOR: Odds ratio adjusted for age, educational level, first-degree family history of PCa, smoking status, body mass index, physical activity, and diabetes mellitus. ^c^ aOR: Odds ratio adjusted for age, educational level, first-degree family history of PCa, smoking status, body mass index, physical activity, diabetes mellitus, and dietary acrylamide intake.

## Data Availability

The data that support the findings of this study are available upon request from the corresponding author.

## Supplementary Materials

The following supporting information can be downloaded at: https://www.mdpi.com/article/10.3390/nu16060836/s1, Table S1: Food groups description and acrylamide concentration for each food; Table S2: Characteristics of controls and prostate cancer (PCa) cases according to tertiles of dietary acrylamide intake in the CAPLIFE study; Table S3: Characteristics of controls and prostate cancer (PCa) cases according to tertiles of acrylamide from acrylamide smoking in the CAPLIFE study; Table S4: Association between the combination of terciles from both sources (diet and cigarettes) and overall prostate cancer (PCa); Figure S1: Approximated nonlinear trend between acrylamide intake (µg/day) and (A) ISUP grade 1–2, (B) ISUP grade 3–5, (C) localized and (D) locally advanced–metastatic prostate cancer (PCa) risk by using restricted cubic spline. Data are odds ratios and 95% confidence interval [aOR (95% CI) adjusted for age, educational level, family history of PCa, smoking status, body mass index, physical activity, diabetes mellitus and energy.
