# Supplementary material for: Dietary and Smoking Acrylamide and Prostate Cancer Risk: CAPLIFE Study
Source: Nutrients. 2024 Mar 14;16(6):836. doi: 10.3390/nu16060836 (PMC10975556; doi:10.3390/nu16060836)
Supplement: Supplementary file 1 [file nutrients-16-00836-s001.zip › nutrients-2878490-supplementary.pdf]

Supplementary Materials

Supplementary Table S1: Food groups description and acrylamide concentration for each food.

Food groups	Food items	Acrylamide (µg/kg)	Source of obtaining the concentration of acrylamide
**Potato fries and chips**	Potato chips	664	[36]
	Fried potato	391	[37]
**Bread**	White cereal<br>Wholemeal bread	42	[10]
**Breakfast cereals**	Cereals	70	[39]
	Whole cereals	152	
**Mixed dishes**	Pasta	13	[10]
	Pizza	40	[37]
	Croquettes	20	[37]
**Cookies, pastries, and chocolates**	Maria cookies	287	[38]
	Whole cookies	448	[38]
	Sponge cake	36	[37]
	Cakes	66	[10]
	Churros	160	[40]
	Chocolates	73	[10]
	Cocoa powder	178	[10]
	Turróna	120	[41]
	Polvoronesb	81	[41]
**Coffee**[c]	Coffee<br>Decaffeinated coffee	256	[42]

[a]Spanish cookies similar to nougat.

[b]Spanish cookies similar to shortbread.

[c]Acrylamide concentration for coffee beverage is expressed in powder equivalent, according to the dilution factor, between 0.035 and 0.125 according to the Scientific Opinion on acrylamide in food of the European Food Safety Authority (EFSA), the average dilution factor (0.08) was applied.

Supplementary Table S2. Characteristics of controls and prostate cancer (PCa) cases according to tertiles of dietary acrylamide intake[1] in the CAPLIFE study.

	Controls			PCa cases		
**Tertiles of dietary acrylamide intake** [a]	**T1**	**T2**	**T3**	**T1**	**T2**	**T3**
	n (%)	n (%)	n (%)	n (%)	n (%)	n (%)
**Total**	131 (33.3)	131 (33.3)	131 (33.3)	138 (32.2)	137 (32.0)	153 (33.8)
**Acrylamide from cigarette smoking** [a] **(µg/day), median (IQR)**	6.2 (0.0-14.0)	8.2 (0.0-14.2)	11.3 (0.0-17.0)	7.8 (0.0-11.3)	9.9 (0.0-15.4)	6.8 (0.0-11.3)
**p-value**		0.438			0.517	
**Age (years), median (IQR)**	69.1 (62.5-73.4)	66.6 (61.0-73.4)	64.6 (60.9-69.9)	70.8 (64.1-75.0)	68.2 (62.4-73.0)	67.0 (62.2-72.7)
**p-value**		0.006			0.022	
**Education, n (%)**						
Primary	28 (21.4)	46 (35.1)	35 (26.7)	48 (34.8)	32 (23.4)	44 (28.8)
Secondary	72 (55.0)	64 (48.9)	63 (48.1)	60 (44.5)	78 (56.9)	90 (58.8)
University	31 (23.6)	21 (16.0)	33 (25.2)	30 (21.7)	27 (19.7)	19 (12.4)
**p-value**		0.085			0.029	
**Marital status, n (%)**						
Married	108 (82.4)	113 (86.3)	109 (83.2)	120 (87.0)	113 (82.5)	123 (80.4)
Not married	23 (17.6)	18 (13.7)	22 (16.8)	18 (13.0)	24 (17.5)	30 (19.6)
**p-value**		0.672			0.316	
**Smoking status, n (%)**						
Never smoker	34 (26.0)	36 (27.5)	33 (25.2)	35 (25.4)	35 (25.6)	42 (27.4)
Former smoker	78 (59.5)	73 (55.7)	68 (51.9)	75 (54.4)	72 (52.6)	84 (54.9)
Current smoker	19 (14.5)	22 (16.8)	30 (22.9)	28 (20.3)	30 (21.9)	27 (17.7)
**p-value**		0.470			0.924	
**Body mass index, n (%)**						
Normal weight	28 (21.4)	23 (17.6)	21 (16.0)	26 (18.8)	27 (19.7)	34 (22.4)
Overweight	69 (52.7)	61 (46.5)	77 (58.8)	73 (52.9)	69 (50.4)	77 (50.6)
Obesity	34 (25.9)	47 (35.9)	33 (25.2)	39 (28.3)	41 (29.9)	41 (27.0)
Missing	-	-	-	-	-	1
**p-value**		0.188			0.937	
**Physical activity, n (%)**						
Low	34 (26.0)	47 (35.9)	52 (39.7)	50 (36.2)	50 (36.5)	66 (43.1)
Moderate	72 (55.0)	65 (49.6)	64 (48.9)	75 (54.4)	73 (53.3)	66 (43.1)
High	25 (19.0)	19 (14.5)	15 (11.4)	13 (9.4)	14 (10.2)	21 (13.8)
**p-value**		0.134			0.308	
**Sedentary behavior (h/day), median (IQR)**	7.0 (5.0-10.0)	7.0 (5.0-9.0)	6.0 (5.0-9.0)	7.0 (6.0-10.0)	7.0 (6.0-9.0)	7.0 (5.0-10.0)
**p-value**		0.302			0.288	
**Energy intake** [b] **(Kcal/day), median (IQR)**	1963.6 (1665.0–2202.9)	2297.4 (1959.3–2658.6)	2912.4 (2454.0–3375.4)	2044.9 (1763.3–2335.1)	2426.2 (2085.1–2898.5)	2870.4 (2489.2–3268.1)
**p-value**		0.001			0.001	

**Alcohol consumption (g/day) [b], median (IQR)**	5.3 (0.7-14.7)	8.8 (2.2-14.8)	7.8 (1.8-18.4)	5.5 (0.7-12.8)	8.8 (1.5-25.6)	9.5 (1.5-20.6)
**p-value**		0.076			0.013	
**Diabetes Mellitus, n (%)**						
No	91 (70.0)	99 (75.6)	109 (83.2)	101 (73.2)	114 (83.2)	122 (80.3)
Yes	39 (30.0)	32 (24.4)	22 (16.8)	37 (26.8)	23 (16.8)	22 (19.7)
Missing	1	-	-	-	-	1
**p-value**		0.042			0.110	
**First–degree family history of PCa, n (%)**						
No	110 (84.0)	119 (90.8)	121 (92.4)	109 (79.0)	105 (76.6)	119 (78.3)
Yes	21 (16.0)	12 (9.2)	21 (7.6)	29 (21.0)	32 (23.4)	33 (21.7)
Missing	-	-	-	-	-	1
**p-value**		0.068			0.890	
**ISUP grade [c], n (%)**						
1–2	-	-	-	103 (75.2)	103 (75.2)	115 (75.2)
3–5	-	-	-	34 (24.8)	34 (24.8)	38 (24.8)
**p-value**					0.999	
**Staging of PCa, n (%)**						
Localized	-	-	-	121 (87.7)	110 (80.3)	138 (90.2)
Locally advanced–metastatic	-	-	-	17 (12.3)	27 (19.7)	15 (9.8)
**p-value**					0.042	

Note: IQR, interquartile range (percentile 25–percentile 75); ISUP: International Society of Urological Pathology.

[a] Acrylamide intake (μg/day) tertiles cut-off points: T1: ≤15.0; T2: >15.0 to ≤24.3; and T3: >24.3.

[b] This information was available for a total of 388 controls and 425 PCa cases.

[c] One subject could not be categorized using ISUP classification as it was a neuroendocrine carcinoma.

Supplementary Table S3. Characteristics of controls and prostate cancer (PCa) cases according to tertiles of acrylamide from smoking in the CAPLIFE study.

	Controls			PCa cases		
**Tertiles from acrylamide smoking** [a]	**T1**	**T2**	**T3**	**T1**	**T2**	**T3**
	n (%)	n (%)	n (%)	n (%)	n (%)	n (%)
**Total**	133 (34.3)	150 (38.7)	105 (27.1)	140 (32.9)	150 (35.3)	135 (31.8)
**Dietary acrylamide intake (µg/day), median (IQR)**	19.2 (13.6-26.4)	17.9 (13.5-31.4)	20.3 (13.0-31.4)	19.3 (13.6-33.5)	19.3 (12.9-32.1)	18.7 (13.8-28.5)
**p-value**		0.621			0.850	
**Age (years), median (IQR)**	67.2 (61.3-72.3)	66.6 (61.0-73.0)	65.9 (61.5-71.5)	69.8 (64.1-75.1)	67.8 (61.0-72.8)	68.0 (62.9-72.1)
**p-value**		0.875			0.040	
**Education, n (%)**						
Primary	32 (24.1)	45 (30.0)	32 (30.5)	41 (29.3)	41 (27.3)	42 (31.1)
Secondary	69 (51.9)	74 (49.3)	51 (48.6)	69 (49.3)	84 (56.0)	73 (54.1)
University	32 (24.0)	31 (20.7)	22 (20.9)	30 (21.4)	25 (16.7)	20 (14.8)
**p-value**		0.780			0.588	
**Marital status, n (%)**						
Married	112 (84.2)	122 (81.3)	91 (86.7)	114 (81.4)	128 (85.3)	111 (82.2)
Not married	21 (15.8)	28 (18.7)	14 (13.3)	26 (18.6)	22 (14.7)	24 (17.8)
**p-value**		0.516			0.643	
**Smoking status, n (%)**						
Never smoker	113 (77.4)	0 (0.0)	0 (0.0)	112 (80.0)	0 (0.0)	0 (0.0)
Former smoker	26 (19.6)	101 (67.3)	89 (84.8)	20 (14.3)	111 (74.0)	98 (72.6)
Current smoker	4 (3.0)	49 (32.7)	16 (15.2)	8 (5.7)	39 (26.0)	37 (27.4)
**p-value**		0.001			0.001	
**Body mass index, n (%)**						
Normal weight	29 (21.8)	28 (18.7)	14 (13.3)	38 (27.3)	26 (17.3)	22 (16.3)
Overweight	73 (54.9)	81 (54.0)	52 (49.5)	64 (46.0)	81 (54.0)	72 (53.3)
Obesity	31 (23.3)	41 (27.3)	39 (37.1)	37 (26.6)	43 (28.7)	41 (30.4)
Missing	-	-	-	1	-	-
**p-value**		0.150			0.163	
**Physical activity, n (%)**						
Low	41 (30.8)	50 (33.3)	40 (38.1)	45 (32.1)	59 (39.3)	60 (44.4)
Moderate	70 (52.6)	73 (48.7)	56 (53.3)	75 (53.6)	78 (52.0)	61 (45.2)
High	22 (16.6)	27 (18.0)	9 (8.6)	20 (14.3)	13 (8.7)	14 (10.4)
**p-value**		0.260			0.202	
**Sedentary behavior (h/day), median (IQR)**	6.0 (5.0-9.0)	7.0 (5.0-9.0)	7.0 (5.0-10.0)	7.0 (5.0-9.0)	7.0 (5.0-10.0)	8.0 (5.0-10.0)
**p-value**		0.188			0.165	

**Energy intake (Kcal/day), median (IQR)**	2207.7 (1932.1–2770.5)	2274.4 (1912.5–2891.2)	2401.9 (1998.8–2960.5)	2465.1 (2082.6–2915.4)	2419.4 (1989.2–2863.9)	2432.6 (2079.5–3076.1)
**p-value**		0.271			0.565	
**Alcohol consumption (g/day), median (IQR)**	5.1 (0.7-12.5)	7.1 (1.5-16.2)	9.4 (1.5-20.7)	5.5 (0.7-12.7)	8.8 (1.4-17.2)	11.0 (2.5-26.0)
**p-value**		0.048			0.002	
**Diabetes Mellitus, n (%)**						
No	103 (77.4)	125 (88.3)	67 (64.4)	120 (86.3)	116 (77.3)	100 (74.1)
Yes	30 (22.6)	25 (16.7)	37 (35.6)	19 (13.7)	34 (22.7)	35 (25.9)
Missing	1	-	-	1	-	-
**p-value**		0.002			0.034	
**First–degree family history of PCa, n (%)**						
No	123 (92.5)	128 (85.3)	95 (90.5)	99 (71.2)	120 (80.0)	112 (83.0)
Yes	10 (7.5)	22 (14.7)	10 (9.5)	40 (28.8)	30 (20.0)	23 (17.0)
Missing	-	-	-	-	-	1
**p-value**		0.136			0.049	
**ISUP grade [b], n (%)**						
1–2	-	-	-	99 (70.7)	114 (76.5)	105 (77.8)
3–5	-	-	-	41 (29.3)	35 (23.5)	30 (22.2)
**p-value**					0.349	
**Staging of PCa, n (%)**						
Localized	-	-	-	114 (81.4)	133 (88.7)	120 (88.9)
Locally advanced–metastatic	-	-	-	26 (18.6)	17 (11.3)	15 (11.1)
**p-value**					0.117	

Note: IQR, interquartile range (percentile 25–percentile 75); ISUP: International Society of Urological Pathology.

[a] Acrylamide from cigarette smoking (µg/day) was available for a total of 388 controls and 425 cases and tertiles cut-off points were: T1: ≤2.3; T2: >2.3 to ≤11.3; and T3: >11.3.

[b] One subject could not be categorized using ISUP classification as it was a neuroendocrine carcinoma.

Supplementary Figure S1. Approximated non-linear trend between acrylamide intake (µg/day) and (A) ISUP grade 1–2, (B) ISUP grade 3–5, (C) localized and (D) locally advanced–metastatic prostate cancer (PCa) risk by using restricted cubic spline. Data are odds ratios and 95 % confidence interval [aOR (95% CI) adjusted for age, educational level, family history of PCa, smoking status, body mass index, physical activity, diabetes mellitus and energy.

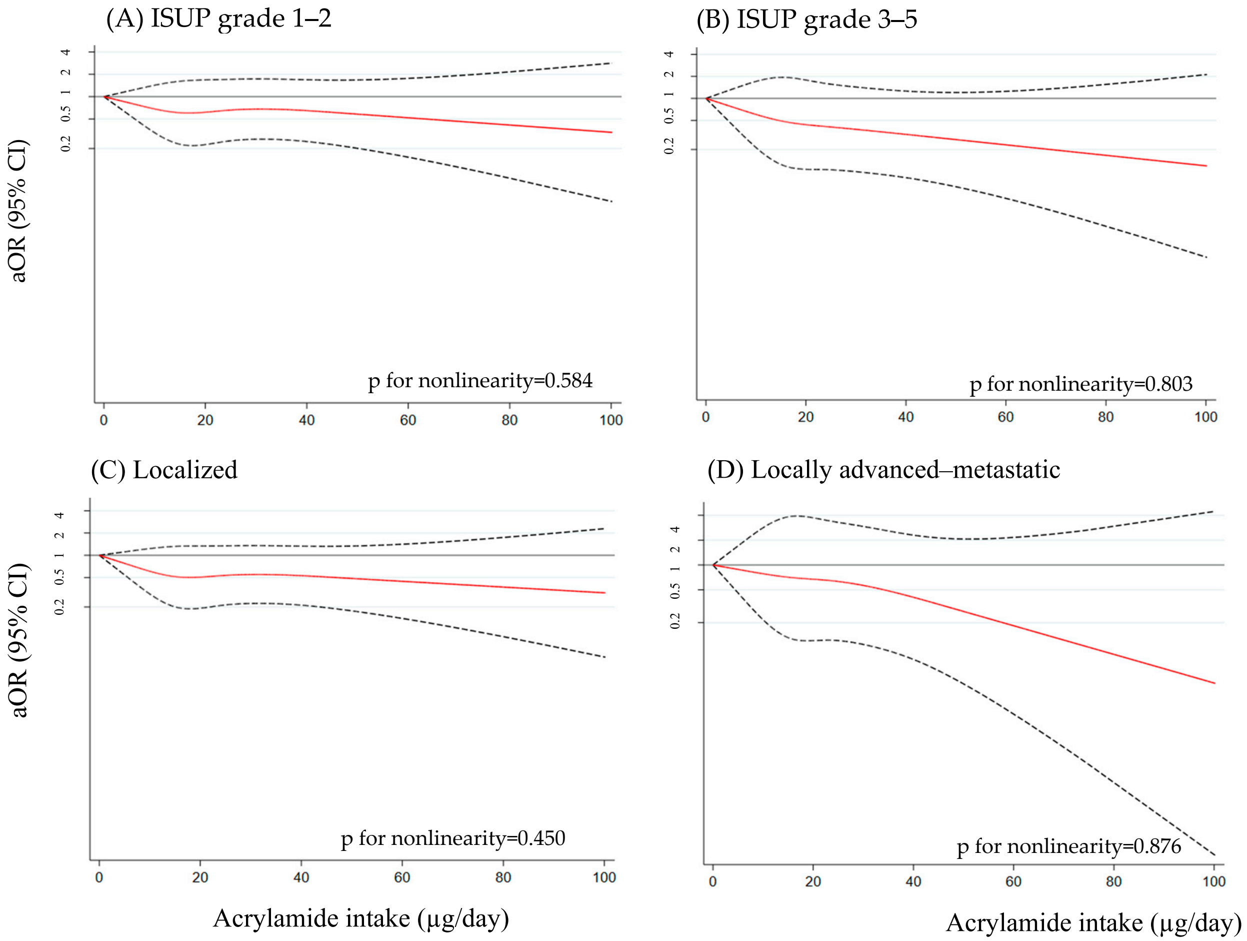

Note: dashed lines indicate the 95% confidence intervals and the red solid line the point estimate of aORs.

Supplementary Table S4: Association between the combination of terciles from both sources (diet and cigarettes) and overall prostate cancer (PCa).

Tertil of dietary acrylamide [a]-Tercil of acrylamide from cigarette smoking [b]	Controls/PCa cases	aOR (95% CI) [c]
T1-T1	46/42	Reference
T1-T2	51/54	1.62 (0.76, 3.46)
T2-T1	45/47	1.14 (0.62, 2.11)
T2-T2	50/40	1.02 (0.47, 2.23)
T3-T1	42/51	1.11 (0.58, 2.12)
T1-T3	33/40	2.00 (0.89, 4.50)
T3-T2	49/56	1.39 (0.63, 3.05)
T2-T3	34/50	2.07 (0.93, 4.60)
T3-T3	38/45	1.52 (0.67, 3.45)
	p-trend	0.172

[a] Acrylamide intake (μg/day) tertiles cut-off points: T1: ≤15.0; T2: >15.0 to ≤24.3; and T3: >24.3.

[b] Acrylamide from cigarette smoking (μg/day) was available for a total of 388 controls and 425 PCa cases and the tertiles cut-off points were: T1: ≤2.3; T2: >2.3 to ≤11.3; and T3: >11.3.

[c] aOR: Odds ratio adjusted for age, educational level, first-degree family history of PCa, smoking status, body mass index, physical activity, diabetes mellitus and total energy.
